# Tuberculosis Is Not a Risk Factor for Primary Biliary Cirrhosis: A Review of the Literature

**DOI:** 10.1155/2012/218183

**Published:** 2012-10-30

**Authors:** Daniel S. Smyk, Dimitrios P. Bogdanos, Albert Pares, Christos Liaskos, Charalambos Billinis, Andrew K. Burroughs, Eirini I. Rigopoulou

**Affiliations:** ^1^Institute of Liver Studies, King's College Hospital and Division of Transplantation Immunology and Mucosal Biology, School of Medicine, King's College London, London SE5 9RS, UK; ^2^Cellular Immunotherapy and Molecular Immunodiagnostics, Center for Research and Technology Thessaly, 41222 Larissa, Greece; ^3^Department of Medicine, University Hospital of Larissa, University of Thessaly School of Medicine, 41110 Larissa, Greece; ^4^Liver Unit, Hospital Clínic de Barcelona, IDIBAPS, CIBERehd, University of Barcelona, 08036 Barcelona, Spain; ^5^Faculty of Veterinary Science, University of Thessaly, 43100 Karditsa, Greece; ^6^The Royal Free Sheila Sherlock Liver Centre, Royal Free Hospital and Department of Surgery, University Collegue London, London NW32QG, UK

## Abstract

Primary biliary cirrhosis (PBC) is a progressive cholestatic liver disease characterised serologically by cholestasis and the presence of high-titre antimitochondrial antibodies, and histologically by chronic nonsuppurative cholangitis and granulomata. As PBC is a granulomatous disease and *Mycobacterium tuberculosis* is the most frequent cause of granulomata, a causal relation between tuberculosis and PBC has been suggested. Attempts to find serological evidence of PBC-specific autoantibodies such as AMA have been made and, conversely, granulomatous livers from patients with PBC have been investigated for molecular evidence of *Mycobacterium tuberculosis*. This paper discusses in detail the reported data in support or against an association between *Mycobacterium tuberculosis* infection and PBC. We discuss the immunological and microbiological data exploring the association of PBC with exposure to *Mycobacterium tuberculosis*. We also discuss the findings of large epidemiologic studies investigating the association of PBC with preexistent or concomitant disorders and the relevance of these findings with tuberculosis. Genome-wide association studies in patients with tuberculosis as well as in patients with PBC provide conclusive hints regarding the assumed association between exposure to this mycobacterium and the induction of PBC. Analysis of these data suggest that *Mycobacterium tuberculosis* is an unlikely infectious trigger of PBC.

## 1. Introduction

Primary biliary cirrhosis (PBC) is a chronic cholestatic, autoimmune liver disease characterised by progressive inflammatory destruction of the small and medium intrahepatic bile ducts and subsequent fibrosis, cirrhosis [1–5], and eventually liver failure [6, 7]. The disease predominantly affects middle-aged women and is practically absent in children or youngsters [8–11]. PBC affects more than one member within the same family, and several reports indicate that first degree relatives of PBC patients have an increased risk of developing the disease [12, 13]. The prevalence of the disease varies among countries, with a recent systematic review indicating prevalence to be 1.91–40.2 per 100,000 inhabitants, and the incidence to be 0.33–5.8 per 100,000 inhabitants/year, in European and North American cohorts (although a breakdown of ethnicity was not provided) [14]. There is a consensus, however, that despite the heterogeneity in the estimated prevalence and incidence amongst ethnic groups, the incidence and prevalence of PBC is increasing [14–18]. The reasons for this increase are poorly understood. Whether there is a true increase or it is due to the awareness for the disease amongst clinicians and the meticulous diagnostic assessment, such a disease-specific autoantibody testing remains to be seen.

Several autoantibody profiles have been found to be specific for the disease [19] and aid in the diagnostic workup of PBC. These include antimitochondrial antibodies (AMA) [20–24] and/or disease-specific antinuclear antibody (ANA) [25–27], which are found in both symptomatic and asymptomatic patients [28]. Most common symptoms at presentation are nonspecific and include fatigue, pruritus, Sicca symptomatology, and arthralgias. Autoimmune rheumatic diseases such as Sjögren's syndrome and systemic sclerosis, as well as other extrahepatic autoimmune manifestations such as autoimmune thyroiditis frequently coexist with PBC [2, 29, 30]. In more severe cases, symptoms relate to portal hypertension and hepatic decompensation (jaundice, ascites, or variceal bleeding), which may indicate the need for liver transplantation [2, 5, 31]. The progression of PBC is generally slow in the majority of the cases [6, 31]. 

The widely accepted diagnostic criteria of PBC consists of three components: (1) biochemical evidence of cholestasis in the form of elevated levels of alkaline phosphatase (ALP) and *γ*GT, (2) seropositivity for disease-specific antimitochondrial antibodies, and (3) histological features on liver biopsy compatible with or diagnostic of PBC [2]. The diagnosis of PBC is probable when at least two of these criteria are met [2, 4]. Most cases have elevated levels of immunoglobulin M (IgM) [2, 4, 32].

The diagnostic hallmark of PBC is the presence of high-titre AMA mainly targeting the E2 subunits of the oxo-acid dehydrogenase complexes (OADC), and in particular that of pyruvate dehydrogenase complex (PDC-E2) [21, 24, 28, 33]. Only 3–10% of patients with PBC lack these antibodies and are considered true AMA-negative PBC patients [28, 34–37]. These autoantibodies are practically nonexistent in patients with other liver diseases unrelated to PBC [38, 39]. Also, true AMA seropositivity in patients with autoimmune rheumatic diseases indicates the current or future development of PBC [23, 40]. Prospective studies have shown that the presence of AMA predicts the future development of PBC in asymptomatic, cholestatic, or acholestatic individuals [22, 41]. ANA specific for the disease can also be present in approximately 50% of patients [42–44]. Published data support the notion that PBC-specific ANA reactivities may have prognostic significance [27, 44–47]. Various other autoantibody specificities have been reported in patients with PBC [48–51]. 

The most prominent histological features of PBC include chronic nonsuppurative cholangitis with or without granulomata demonstrating destruction of biliary epithelial cells and loss of small bile ducts with portal inflammatory cell infiltration (see below) [2, 4, 5]. Genetic factors and environmental triggers have been considered important for the induction of autoimmune disease, such as PBC [8, 52–57]. Several infectious and noninfectious triggers have been implicated in the pathogenesis of PBC [8, 12, 52, 55, 58–64]. 

Because *Mycobacterium tuberculosis *(*M. tuberculosis*) [65] is the most frequent cause of granulomas [66], and in view of reports indicating serological evidence of PBC-specific AMA in patients with tuberculosis, it has been suggested that tuberculosis/*M. tuberculosis* may be a cause of PBC (Table 1). Indeed, *M. tuberculosis *has been indicated as a potential cause of other autoimmune disease such as systemic lupus erythematosus (SLE) [67–74]. This paper will critically analyse the epidemiological, clinical, immunological, and experimental data in support or against the notion that tuberculosis and PBC may be pathogenetically linked.

## 2. Granulomas in PBC

 Up to 15% of liver biopsy material contains evidence of granulomas [82–86]. An analysis of 12,161 liver biopsies revealed the presence of granulomas in 442 (3.6%) [87] cases, including 215 diagnosed with PBC, representing 48.7% of all biopsies with granulomas and 1.8% of all biopsies analysed [87]. Molecular evidence of infectious agents by PCR was found in just 15 samples (3.4%) and *M. tuberculosis* has been detected in three of the 15 (20%) but it is not clear whether the *M. tuberculosis*-positive cases were from livers of patients with PBC [87]. The fact that three or less of the 215 granulomatous livers from PBC cases had molecular evidence of *M. tuberculosis* argues against the notion that this mycobacterium is a trigger for the development of this enigmatic disease. Other studies have also noted and reported a diagnosis of PBC in 24–62% of livers with evidence of granulomas [82, 83, 88]. However, only a small proportion of the PBC granulomata had evidence of mycobacteria, again pointing towards the lack of an association of *M. tuberculosis* with PBC.

Irrespective of the relation of PBC granulomas with *M. tuberculosis* infections, attempts have been made by researchers to delineate the role of immunity in the development of PBC granulomas. A recent comprehensive immunohistochemical analysis by You et al. [89] has shown that CD11c, the classical dendritic cell marker, is highly expressed in PBC granulomas. It also appears that CD11c-positive epitheliod granulomas are more prevalent in patients with PBC at early disease stages. Finally, the expression of CD11c in PBC granulomas is associated with higher IgM levels. These findings further support the notion that the influence of antigenic stimulation to professional antigen presenting cells largely influences the formation of granulomas in PBC. You et al. [89] suggested that PBC liver granulomas may result from the interaction between immature dendritic cells and IgM, but this needs to be addressed at the experimental level. On the other hand, in human tuberculosis the formation of granulomas is considered either as an attempt to contain *M. tuberculosis* infection or an early effort by the pathogenic mycobacteria to assist the spreading of bacteria to uninfected macrophages that are recruited at the site of inflammation [66, 90]. This perception raises the notion that granulomas in human tuberculosis may have a beneficial or catastrophic potential for the host depending on the timing of their formation [91, 92]. 

### 2.1. Evidence of Mycobacterial Infection in PBC

Tanaka et al. searched for evidence of infectious agents by PCR in livers tissues from 29 patients with PBC. Mycobacterial DNA was not detected in any of these samples [93]. Other attempts to provide evidence of mycobacteria DNA in livers from PBC cases have provided inconclusive data. Broome et al. [75] demonstrated positive staining for mycobacterial hsp65 using a monoclonal antibodies in nine of the ten PBC cases [75]. O'Donohue and colleagues [94] studied liver biopsy specimen from eleven PBC cases. Five lymph nodes from patients with tuberculous lymphadenopathy were used as positive controls [94]. Three of the positive controls also had liver biopsies taken for concurrent tuberculous hepatitis. Four of the five positive controls had detectable mycobacterial DNA. Mycobacterial DNA was undetectable in the tissues obtained from patients with PBC [94]. 

### 2.2. PBC-Specific Autoantibodies in Patients with Tuberculosis

An early study by Klein et al. [81] reported the presence of PBC-specific AMA in 12/28 (43%) of patients with tuberculosis. Immunoblotting demonstrated that these sera recognized PDC-E2 based on purified mitochondrial fraction derived from beef heart mitochondrial as an antigenic source. Only 2% of sera from individuals with other viral and bacterial infections showed reactivity with PDC-E2, and there was no reaction with sera from healthy controls [81]. As well, the titres of anti-PDC-E2 antibodies were low [81]. Additionally, none of the anti-PDC-E2 antibody positive sera (1/10 dilution) gave an immunofluorescent pattern typical of PBC by indirect immunofluorescence [19, 21, 22, 81]. This contrasts to what is normally seen in most patients with PBC, where titers of PDC-E2 targeting AMA give typical and strong immunofluorescent staining at dilutions as high as 1/100,000. Amongst the 12 AMA positive cases with active tuberculosis, six had abnormal levels of *γ*GT and four had elevated levels of alkaline phosphatase, with only two of the twelve cases having increased IgM levels. None of the patients had increased bilirubin levels or evidence of impaired synthetic function. Liver biopsies were not performed and it is not known what has happen to these individuals over the years, and whether they have developed clinically overt liver disease. There was no information as to whether other causes of abnormal liver biochemistry such as drug-induced hepatotoxicity were excluded.

Patients with *M. tuberculosis* and AMA positivity show reactivity to PDC-E2, which has not been observed in those infected with other forms of mycobacteria [81, 95]. Gilburd et al. [95] suggested sequence homology between *M. leprae* antigens and the 35, 41, and 54 kDa subunits of OADC, implying that molecular mimicry and immunological cross-reactivity between *M. leprae* and human mitochondrial autoantigens may be responsible for the induction of AMA in patients with leprosy. 

### 2.3. Mycobacteria and PBC: The Role of Molecular Mimicry

It is unclear as to whether a negative result for *M. tuberculosis* also rules out the effects of a previous and nonactive infection. It has been suggested that mycobacteria and other infectious agents may be involved in the initiation of autoimmunity by microbial/self-immunological cross-reactivity, where infection and clearance of the infection occur before the onset of clinical disease [96–100]. The role of molecular mimicry has been studied by several groups [96, 101–107] and immunological cross-reactivity has been documented [108–113] as a mechanism responsible for the induction of autoantigen-specific immune responses in microbial-triggered autoimmunity [107, 114–117] in susceptible individuals [118, 119]. Impairment in the immunosuppressory functions of the host appears to be important for the induction and perpetuation of autoaggression induced via molecular mimicry or other mechanisms [120, 121]. Indeed, our group has studied molecular mimicry as a potential inducer of autoimmunity in several autoimmune liver and gastrointestinal diseases [97, 104, 110, 122, 123]. Most of the microbial/self-homologues are not targets of cross-reactive responses, and this underlines the importance of disease-specific pairs targeted by antibodies [99, 100, 111, 113, 124, 125]. Of relevance to mycobacteria, Vilagut et al. [126] has reported that hsp65 kDa *M. gordonae* and human OADC antigens, including human PDC-E2, are cross-reactive [127]. They also reported the presence of mycobacterial DNA in livers from patients with PBC. We have attempted to better define the extent of this cross-reactivity at the peptidyl level and through amino-acid comparison database searches, and we have identified an amino-acid homology between human PDC-E2, and mycobacterial hsp65 [124]. Thus, the hexameric motif [GDL(IL)AE)] is present in hsp65_94–99_ and the major PBC-specific mitochondrial autoepitopes, namely, the inner lipoyl PDC-E2_216–221_ and the outer lipoyl domain human PDC-E2_102–107_ [124]. The hsp65 mimic was not restricted to *M. gordonae*, but was conserved amongst mycobacteria (including *M. tuberculosis*). The SxGDL[IL]AE motif is virtually unique to mycobacterial hsp, and human PDC-E2 is the only known human sequence containing it [124]. We also obtained data to suggest that peptides spanning the homologous mycobacterial hsp65 and human PDC-E2 sequences are targets of cross-reactive responses when serum samples from patients with PBC are tested. These data support the notion that mycobacterial infection could initiate antimycobacterial responses against hsp65, which in turn could cross-react with human PDC-E2. The relevance of the biological significance of these data in the immunopathogenesis of microbial-induced PBC remains unaddressed. 

If there is a link between tuberculosis and PBC, it would be expected that areas where *M. tuberculosis* is endemic would have high rates of PBC, but this is not the case. For example, southern Africa is endemic for tuberculosis, but PBC is relatively rare in those regions [128, 129]. The same could be said for India and China, which have relatively high rates of tuberculosis but low rates of PBC [130–132]. Also, epidemiological studies assessing susceptibility to PBC would be expected to identify tuberculosis as a risk factor. 

## 3. Risk Factors of PBC: Epidemiological Studies

 Several epidemiological studies have been conducted to investigate risk factors for the development of PBC [77–80]. These studies have largely been based on questionnaires addressing geographical and lifestyle factors, as well as personal and familial medical and surgical histories. However, very few of these studies specifically mention infection with *M. tuberculosis. *A study by Parikh-Patel and colleagues [79] administered a standardized US National Health and Nutrition Examination Study (NHANES) questionnaire to 241 PBC patients from the USA, in addition to 261 of their siblings and 141 friends as controls. Within the medical histories, it was found that 2 patients with PBC (1.2%) reported having tuberculosis as adults, compared to none of the siblings, and one friend (0.8%) [79]. A larger study conducted by Gershwin et al. [78] was also based on an NHANES questionnaire. The cohort in that study consisted of 1032 PBC patients from 23 tertiary care centres in the USA, 1041 controls selected from a random-digit-dialling protocol, which were sex, age, race, and geographically matched. All participants were administered the questionnaire by trained professionals, but it was not indicated as to whether specific questions were asked regarding tuberculosis [78]. However, it appears that questions were raised as to whether participants had been vaccinated for tuberculosis [78]. Despite this, it does not appear that any significant link was found in regards to tuberculosis and/or tuberculosis infection [78]. Prince et al. [80] conducted an epidemiological study involving 318 PBC patients from an epidemiological study and 2258 from a PBC support group, in addition to 2438 controls. There was no indication as to whether specific questions were raised in regards to tuberculosis [80]. Corpechot and colleagues [77] administered a standardized questionnaire to 222 PBC patients and 509 age, sex, and residentially matched controls [77]. Although no history of previous tuberculosis infection was indicated, it appears that questions regarding immunization for tuberculosis were asked, as 1% of PBC cases reported vaccination, although 1% of controls also reported vaccination [77]. Based on the epidemiological evidence, it does not appear that infection with *M. tuberculosis* is linked with PBC. However, it should also be noted that it is not clear as to whether history of tuberculosis infection and/or vaccination was investigated in some studies. In those which do, there was no significantly higher incidence of PBC infection among patients compared to controls, and there was no significantly higher incidence of vaccination against tuberculosis in PBC patients. An association between vaccination and the development of PBC has been reported for other microbial agents, and in particular Lactobacilli, and the mechanism of molecular mimicry has been proposed to account for the induction of PBC-specific autoreactivity [97, 98]. 

## 4. Case Studies of PBC and Tuberculosis

A PubMed search for case reports in of patients with tuberculosis who developed PBC (search terms: “primary biliary cirrhosis, tuberculosis” and “primary biliary cirrhosis, *mycobacterium tuberculosis*”) revealed one case [76]. That case reports of a 70-year-old Tunisian patient who developed antimitochondrial antibodies and anti-ADN during urogenital tuberculosis. There were clinical and biological signs of primary biliary cirrhosis and systemic lupus erythematosus in that patient [76]. If there was a link between tuberculosis and PBC, it would be expected that several case reports of patients developing PBC, or AMA positivity following infection with *M. tuberculosis*.

## 5. Genome-Wide Association Studies in Tuberculosis and in PBC

Genome-wide association studies (GWAS) have recently shed light on the genetic background of PBC and pulmonary tuberculosis. Genes implicated in PBC lay within both HLA and non-HLA regions (Table 2) [133–140]. GWAS have led to the identification of several genes that infer susceptibility to developing active pulmonary tuberculosis l [141, 142] including JAG1, DYNLRB2, EBF1, TMEFF2, CCL17, HAUS6, PENK, and TXNDC4. If tuberculosis is a risk factor for the development of PBC, it would be reasonable to infer that both conditions would share several susceptibility genes [143]. However, no significant overlapping of genes conferring susceptibility to both PBC and tuberculosis has been identified (Table 2). Also depending on the geographical region, environmental exposures related to tuberculosis may differ, as well as strong associations between different evolutionary lineages of *Mycobacterium tuberculosis *with specific geographical regions have been noted [144]. Such differences have been extensively studied in tuberculosis patients, such as those originating from Indonesian patients living in Jakarta and Bandung compared to other regions/islands. The extent by which genetic co-evolution and phylogenetic difference involving *M. tuberculosis* participate in the dysregulation of host's immune response is worthy for further investigation.

## 6. Tuberculosis in Other Autoimmune Diseases

PBC is not the only autoimmune disease in which *M. tuberculosis* has been implicated. *M. Tuberculosis* has also been noted to be involved in a more common autoimmune disease, SLE, and specifically to disease flares. An early study by Shoenfeld and colleagues [73] examined whether murine monoclonal anti-TB antibodies reacted with ssDNA, dsDNA, and other polynucleotides and found that monoclonal anti-DNA autoantibodies from humans and mouse SLE models bound to three glycolipids shared among mycobacteria. Incubation of the antibodies with ssDNA and/or polynucleotides or glycolipid antigens was found to inhibit binding [73]. This suggests a possible explanation for the production of autoantibodies in mycobacterial infections. Sela and colleagues [145] examined the sera from 57 patients with untreated TB for the anti-DNA idiotype 16/6 and found that 60% had increased levels of the idiotype compared to 4% of controls. As well, there were also increased levels of autoantibodies such as those to ssDNA, dsDNA, polynucleotides, and cardiolipin [145]. A study by Amital-Teplizki et al. [67] examined the binding of human lupus anti-DNA antibodies and murine antimycobacterial antibodies to human cortical brain tissue. That group found that antimycobacterial and anti-DNA antibodies competed on the binding site of a common neuronal membrane epitope, indicating the presence of a shared antigen between mycobacteria and DNA [67]. 

In addition to the above molecular findings, a link between SLE flares and TB have also been noted. Ribeiro and colleagues [74] describe the development of TB infection in parallel with lupus flares in four females. The site of mycobacterial infection/detection in the women included a positive urine culture for mycobacterial spp., a nodular pulmonary lesion with subsequent positive tuberculin cutaneous test, a positive acid-fact bacilli test on bronchoalveolar lavage, and one case of disseminated TB [74]. In all four women, SLE flares did not improve on administration of antirheumatic treatment and immunosuppression but did improve after the administration of antituberculosis treatment [74]. Those authors suggest that *M. tuberculosis* stimulated the production of autoantibodies with shared affinity for mycobacterial and human antigens, which in turn may have led to the SLE flare [74]. These cases indicate a possible link between SLE and TB, but it should also be noted that the immunosuppressive treatment given to SLE patients may in fact reactivate a latent TB infection. 

## 7. Conclusion

The study of the role of *Mycobacterium tuberculosis* in PBC has been limited due to the scarcity of studies and data. Multiple epidemiological studies have failed to indicate tuberculosis as a risk factor for the development of PBC, and there is an absence of case reports of tuberculosis infection followed by PBC in the literature. It would be expected that if *M. tuberculosis* was linked with PBC, there would be a higher incidence of PBC in areas which are endemic for tuberculosis. However, this does not appear to be the case [146]. It would also be expected that if *M. tuberculosis* was linked to PBC, several genes which predispose to one disease, would overlap with the other. However, with the advent of GWAS, this link has also been ruled out, with no genes being shared between the two conditions. Although more studies are needed to investigate a potential link, the existent data are highly suggestive of a lack of a causative link between tuberculosis and PBC. However, the observation of granulomatous lesion in PBC liver biopsies warrants further investigation, as the significance of these lesions in the liver of PBC patients is unknown.

## Figures and Tables

**Table 1:** Summary of the major findings of studies investigating the role of *Mycobacterium tuberculosis* as a trigger of PBC. The findings are presented in relation to Koch's postulates. To date, no conclusive evidence has been presented which links *Mycobacterium tuberculosis* with PBC.

Koch's postulates	Finding	Study
*Infectious aetiology* Microorganism must be present in every case of diseased individuals	Positive staining for mycobacterial hsp65 in PBC cases has been demonstrated, but hsp65 is conserved among all mycobacterial species and it is unclear whether this staining is *Mycobacterium tuberculosis-*specific and characteristic for PBC patients with granulomata	[75]
*Isolation* The microorganism must be isolated and grown in pure cultures	No such data exist	
*Disease causality* Pure cultures inoculated in healthy animals must reproduce the disease	No such data exist Only one case report of a female that developed PBC following tuberculosis infection has been published There is no epidemiological or evidence linking tuberculosis with PBC, including vaccination against tuberculosis	[76] [77–80]
*Reproducibility* The microorganism must be recovered from the diseased animal	No such data exist	
*Other data indirectly relevant to Koch's postulates *	PBC specific AMA was detected in 43% of tuberculosis patients, but in low titres, and did not show the typical indirect immunofluorescent patterns of antimitochondrial antibodies seen in PBC. These patients do not have clinical features of PBC.	[19, 21, 22, 81]

**Table 2:** Major susceptibility genes associated with tuberculosis [141, 142] and primary biliary cirrhosis [133–140], as reported through genome wide association studies. Note that no positive associations are significantly shared between primary biliary cirrhosis (PBC) and pulmonary tuberculosis (TB).

Gene	PBC	TB
HLA		
DR8	+	−
DQB1	+	−
DRB1	+	−
DQA1	+	−
DQA2	+	−
Non-HLA		
STAT4	+	−
SPIB	+	−
IRF5	+	−
IL12A	+	−
IL12RB	+	−
MMEL1	+	−
CXCR5	+	−
NFKB1	+	−
JAG1	−	+
DYNLRB2	−	+
EBF1	−	+
TMEFF2	−	+
CCL17	−	+
HAUS6	−	+
PENK	−	+
TXNDC4	−	+
